# Di-*tert*-butyl 1-[2-hy­droxy-3-(methyl­sulfan­yl)prop­yl]hydrazine-1,2-di­carboxyl­ate

**DOI:** 10.1107/S1600536814015062

**Published:** 2014-07-02

**Authors:** Xiao-Guang Bai, Xiao-Yu Yang, Ju-Xian Wang

**Affiliations:** a Institute of Medicinal Biotechnology, Chinese Academy of Medical Sciences and Peking Union Medical College, Beijing 100050, People’s Republic of China; b HangDeXin (Beijing) Pharmatech. Co. Ltd, Fengtai District, Beijing 100050, People’s Republic of China

**Keywords:** crystal structure

## Abstract

The title compound, C_14_H_28_N_2_O_5_S, was synthesized by the reaction of 2-[(methyl­sulfan­yl)meth­yl]oxirane with di-*tert*-butyl oxalate in hydrazine hydrate. In the crystal, mol­ecules are linked by N—H⋯O and O—H⋯O hydrogen bonds into supra­molecular chains propagating along the *b*-axis direction.

## Related literature   

For the synthesis of the title compound, see: Budavari *et al.* (1989 ▶); Mendling *et al.* (2002 ▶).
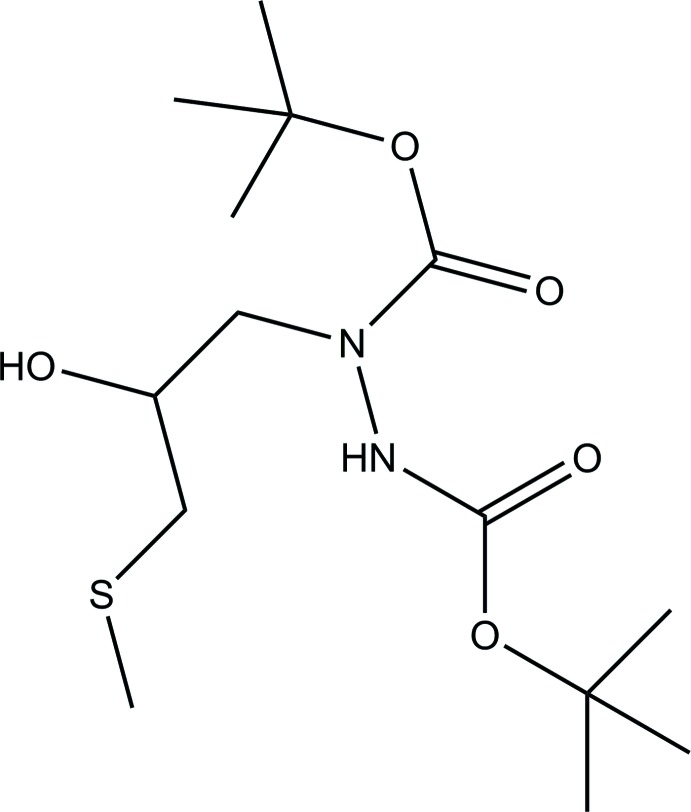


## Experimental   

### 

#### Crystal data  

C_14_H_28_N_2_O_5_S
*M* *_r_* = 336.44
Monoclinic, 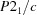

*a* = 14.0172 (3) Å
*b* = 7.83649 (15) Å
*c* = 17.2076 (3) Å
β = 103.772 (2)°
*V* = 1835.84 (7) Å^3^
*Z* = 4
Cu *K*α radiation
μ = 1.77 mm^−1^
*T* = 293 K
0.28 × 0.24 × 0.24 mm

#### Data collection  

Agilent Xcalibur (Atlas, Gemini ultra) diffractometer
Absorption correction: multi-scan (*CrysAlis PRO*, Agilent, 2013 ▶) *T*_min_ = 0.551, *T*_max_ = 0.680
16825 measured reflections
3247 independent reflections
2903 reflections with *I* > 2σ(*I*)
*R*_int_ = 0.028

#### Refinement  

*R*[*F* ^2^ > 2σ(*F* ^2^)] = 0.063
*wR*(*F* ^2^) = 0.187
*S* = 1.00
3247 reflections
207 parameters
H-atom parameters constrained
Δρ_max_ = 1.02 e Å^−3^
Δρ_min_ = −0.66 e Å^−3^

### 

Data collection: *CrysAlis PRO* (Agilent, 2013 ▶); cell refinement: *CrysAlis PRO*; data reduction: *CrysAlis PRO*; program(s) used to solve structure: *SHELXS97* (Sheldrick, 2008 ▶); program(s) used to refine structure: *SHELXL97* (Sheldrick, 2008 ▶); molecular graphics: *SHELXTL/PC* (Sheldrick, 2008 ▶); software used to prepare material for publication: *SHELXTL/PC*.

## Supplementary Material

Crystal structure: contains datablock(s) I, New_Global_Publ_Block. DOI: 10.1107/S1600536814015062/xu5797sup1.cif

Structure factors: contains datablock(s) I. DOI: 10.1107/S1600536814015062/xu5797Isup2.hkl

Supporting information file. DOI: 10.1107/S1600536814015062/xu5797Isup3.cml

CCDC reference: 1010387

Additional supporting information:  crystallographic information; 3D view; checkCIF report

## Figures and Tables

**Table 1:** Hydrogen-bond geometry (Å, °)

*D*—H⋯*A*	*D*—H	H⋯*A*	*D*⋯*A*	*D*—H⋯*A*
O5—H5⋯O2^i^	0.82	2.03	2.842 (3)	168
N2—H2⋯O5^i^	0.93	2.07	2.996 (3)	172

Symmetry code: (i) 
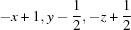
.

**Fig. 1.**
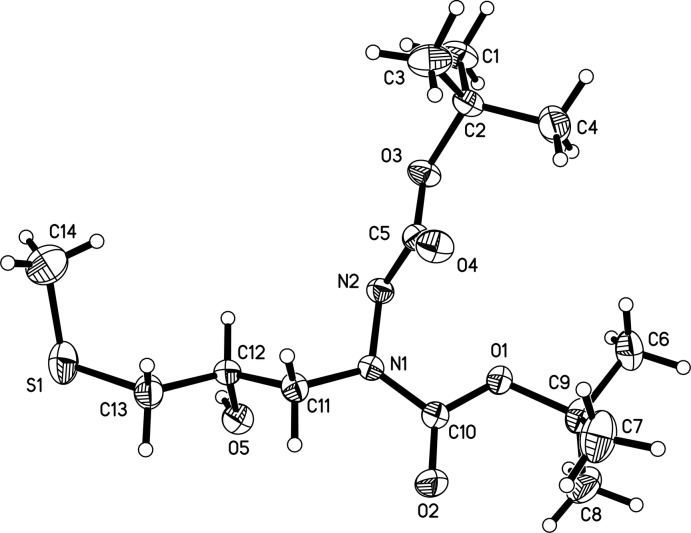
The molecular structure of the title compound with displacement ellipsoids drawn at the 30% probability level.

**Fig. 2.**
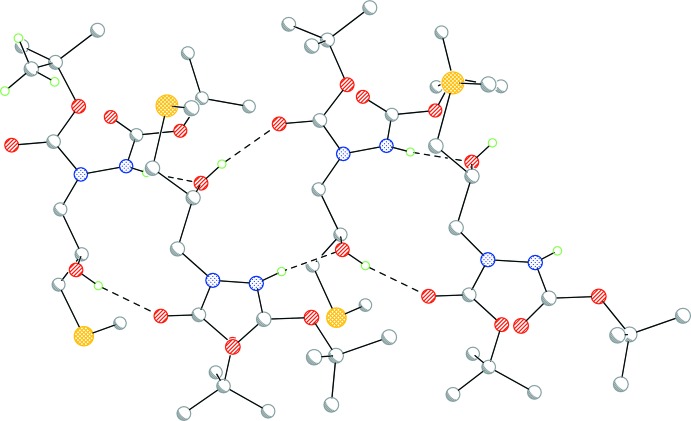
View of the one-dimensional chains of the title compound extending along the b axis. All the hydrogen atoms except those involved in hydrogen bonding have been omitted for clarity. Hydrogen bonds are shown as dashed lines.

## supplementary crystallographic information

### S1. Comment

Nifuratel is a medicine used in gynecology (Budavari *et al.*, 1989; Mendling *et al.*, 2002). It is a local antiprotozoal and antifungal agent that may also be given orally. The tile compound is a key intermediate of nifuratel, herewith we report the synthesis and the crystal structure of the title compound. In the molecule of the title compound, all bond lengths and angles have normal values with C—C bond lengths between 1.510 (4) to 1.514 (4) Å and slightly shorter C—N distances, 1.367 (3) and 1.460 (3) Å, as expected (Fig. 1). Molecules are linked by N5—H5···O2^i^ and N2—H2···O5^i^ (i = -*x* + 1, *y* - 1/2, -*z* + 1/2) hydrogen bonds involving the imino group N atom, the ester group O atom and hydroxyl O atom into chains running parallel to the *b* axis (Fig. 2).

### S2. Experimental

In a 500 ml four-necked round-bottom flask equipped with a mechanical stirrer 2-((methylsulfanyl)methyl)oxirane (55.8 g) was cautiously dissolved in 80% hydrazine hydrate (17.5 g). The solution was heated at 95°C for 6 h, then the hydrazine hydrate was removed by reduced pressure distillation at 85°C. 125 ml me thanol and Boc_2_O was added into the remaining aqueous phase group by group. The reaction was completion after 2 h, the crude product were obtained. The crude product was purified by column chromatography to obtain 44.3 g (78.8%) of product which was recrystallized from 200 ml of a mixture of methanol and acetone (*v*/*v* = 1/2) to yield 25.3 g (57%) of clear light colourless block-like crystals.

### S3. Refinement

H atoms were placed in calculated positions and refined constrained to ride on their parent atoms, with C—H = 0.96—0.97 Å, N—H = 0.93 Å and O—H = 0.82, *U*_iso_(H) = 1.5*U*_eq_(C,O) for methyl and hydroxyl H atoms and 1.2*U*_eq_(C,N) for the others.

### Crystal data

**Table:**

C_14_H_28_N_2_O_5_S	*F*(000) = 728
*M**_r_* = 336.44	*D*_x_ = 1.217 Mg m^−^^3^
Monoclinic, *P*2_1_/*c*	Cu *K*α radiation, λ = 1.54184 Å
Hall symbol: -P 2ybc	Cell parameters from 7294 reflections
*a* = 14.0172 (3) Å	θ = 5.3–66.4°
*b* = 7.83649 (15) Å	µ = 1.77 mm^−^^1^
*c* = 17.2076 (3) Å	*T* = 293 K
β = 103.772 (2)°	Block, colourless
*V* = 1835.84 (7) Å^3^	0.28 × 0.24 × 0.24 mm
*Z* = 4	

### Data collection

**Table:**

Agilent Xcalibur (Atlas, Gemini ultra) diffractometer	3247 independent reflections
Radiation source: fine-focus sealed tube	2903 reflections with *I* > 2σ(*I*)
Graphite monochromator	*R*_int_ = 0.028
ω scans	θ_max_ = 66.6°, θ_min_ = 3.3°
Absorption correction: multi-scan (*CrysAlis PRO*, Agilent, 2013)	*h* = −16→16
*T*_min_ = 0.551, *T*_max_ = 0.680	*k* = −9→7
16825 measured reflections	*l* = −20→20

### Refinement

**Table:**

Refinement on *F*^2^	Primary atom site location: structure-invariant direct methods
Least-squares matrix: full	Secondary atom site location: difference Fourier map
*R*[*F*^2^ > 2σ(*F*^2^)] = 0.063	Hydrogen site location: inferred from neighbouring sites
*wR*(*F*^2^) = 0.187	H-atom parameters constrained
*S* = 1.00	*w* = 1/[σ^2^(*F*_o_^2^) + (0.1098*P*)^2^ + 1.6106*P*] where *P* = (*F*_o_^2^ + 2*F*_c_^2^)/3
3247 reflections	(Δ/σ)_max_ < 0.001
207 parameters	Δρ_max_ = 1.02 e Å^−^^3^
0 restraints	Δρ_min_ = −0.66 e Å^−^^3^

### Special details

**Table:**

Geometry. All e.s.d.'s (except the e.s.d. in the dihedral angle between two l.s. planes) are estimated using the full covariance matrix. The cell e.s.d.'s are taken into account individually in the estimation of e.s.d.'s in distances, angles and torsion angles; correlations between e.s.d.'s in cell parameters are only used when they are defined by crystal symmetry. An approximate (isotropic) treatment of cell e.s.d.'s is used for estimating e.s.d.'s involving l.s. planes.
Refinement. Refinement of *F*^2^ against ALL reflections. The weighted *R*-factor *wR* and goodness of fit *S* are based on *F*^2^, conventional *R*-factors *R* are based on *F*, with *F* set to zero for negative *F*^2^. The threshold expression of *F*^2^ > σ(*F*^2^) is used only for calculating *R*-factors(gt) *etc*. and is not relevant to the choice of reflections for refinement. *R*-factors based on *F*^2^ are statistically about twice as large as those based on *F*, and *R*- factors based on ALL data will be even larger.

### Fractional atomic coordinates and isotropic or equivalent isotropic displacement parameters (Å^2^)

**Table:**

	*x*	*y*	*z*	*U*_iso_*/*U*_eq_	
C1	0.8328 (2)	0.0844 (4)	0.2221 (2)	0.0607 (8)	
H1A	0.8060	0.0494	0.2660	0.091*	
H1B	0.8898	0.0169	0.2216	0.091*	
H1C	0.7845	0.0688	0.1728	0.091*	
C2	0.86149 (18)	0.2704 (3)	0.23178 (17)	0.0442 (6)	
C3	0.8960 (3)	0.3336 (4)	0.1599 (2)	0.0654 (9)	
H3A	0.8445	0.3187	0.1125	0.098*	
H3B	0.9527	0.2697	0.1551	0.098*	
H3C	0.9127	0.4523	0.1667	0.098*	
C4	0.9360 (2)	0.3034 (4)	0.3097 (2)	0.0661 (8)	
H4A	0.9505	0.4232	0.3147	0.099*	
H4B	0.9951	0.2410	0.3102	0.099*	
H4C	0.9097	0.2670	0.3536	0.099*	
C5	0.75898 (17)	0.5211 (3)	0.24304 (13)	0.0377 (5)	
C6	0.8608 (3)	0.5688 (5)	0.4950 (2)	0.0734 (10)	
H6A	0.8161	0.4772	0.4973	0.110*	
H6B	0.9031	0.5863	0.5471	0.110*	
H6C	0.8996	0.5405	0.4578	0.110*	
C7	0.8696 (3)	0.8736 (6)	0.4540 (2)	0.0829 (12)	
H7A	0.8977	0.8445	0.4100	0.124*	
H7B	0.9212	0.8901	0.5013	0.124*	
H7C	0.8322	0.9769	0.4418	0.124*	
C8	0.7408 (3)	0.7799 (5)	0.52474 (19)	0.0717 (9)	
H8A	0.7031	0.8797	0.5050	0.107*	
H8B	0.7824	0.8035	0.5766	0.107*	
H8C	0.6972	0.6876	0.5288	0.107*	
C9	0.8032 (2)	0.7309 (4)	0.46795 (15)	0.0502 (7)	
C10	0.68395 (18)	0.7871 (3)	0.34172 (15)	0.0398 (6)	
C11	0.59483 (19)	0.8209 (3)	0.20202 (15)	0.0420 (6)	
H11A	0.6411	0.8357	0.1687	0.050*	
H11B	0.5821	0.9327	0.2217	0.050*	
C12	0.50032 (19)	0.7509 (3)	0.15139 (15)	0.0414 (6)	
H12	0.5117	0.6371	0.1318	0.050*	
C13	0.4655 (2)	0.8726 (4)	0.08046 (17)	0.0540 (7)	
H13A	0.5206	0.8962	0.0571	0.065*	
H13B	0.4464	0.9797	0.1007	0.065*	
C14	0.4265 (4)	0.6873 (6)	−0.0605 (3)	0.1003 (15)	
H14A	0.4392	0.7640	−0.1004	0.150*	
H14B	0.3861	0.5948	−0.0862	0.150*	
H14C	0.4875	0.6430	−0.0294	0.150*	
N1	0.63988 (15)	0.7134 (3)	0.27015 (12)	0.0406 (5)	
N2	0.66615 (14)	0.5497 (2)	0.25167 (12)	0.0384 (5)	
H2	0.6317	0.4615	0.2686	0.046*	
O1	0.73806 (14)	0.6746 (2)	0.39125 (10)	0.0453 (4)	
O2	0.67196 (16)	0.9354 (2)	0.35674 (12)	0.0576 (5)	
O3	0.76770 (12)	0.3515 (2)	0.23478 (12)	0.0450 (5)	
O4	0.81944 (14)	0.6293 (2)	0.24135 (12)	0.0512 (5)	
O5	0.43100 (14)	0.7407 (2)	0.19925 (12)	0.0496 (5)	
H5	0.3943	0.6593	0.1847	0.074*	
S1	0.36497 (6)	0.79803 (14)	0.00276 (5)	0.0743 (3)	

### Atomic displacement parameters (Å^2^)

**Table:**

	*U*^11^	*U*^22^	*U*^33^	*U*^12^	*U*^13^	*U*^23^
C1	0.0588 (17)	0.0400 (15)	0.087 (2)	0.0079 (13)	0.0240 (16)	−0.0047 (14)
C2	0.0374 (13)	0.0403 (13)	0.0560 (15)	0.0054 (10)	0.0135 (11)	−0.0014 (11)
C3	0.071 (2)	0.0624 (19)	0.074 (2)	−0.0013 (16)	0.0392 (17)	−0.0057 (16)
C4	0.0560 (18)	0.0638 (19)	0.070 (2)	0.0053 (15)	−0.0009 (15)	−0.0018 (16)
C5	0.0417 (12)	0.0355 (12)	0.0348 (11)	0.0021 (10)	0.0067 (9)	−0.0013 (9)
C6	0.064 (2)	0.090 (3)	0.0553 (18)	0.0143 (18)	−0.0082 (15)	0.0067 (17)
C7	0.067 (2)	0.107 (3)	0.069 (2)	−0.031 (2)	0.0045 (17)	0.004 (2)
C8	0.080 (2)	0.091 (3)	0.0456 (16)	−0.0027 (19)	0.0186 (16)	−0.0128 (16)
C9	0.0487 (15)	0.0645 (17)	0.0341 (12)	−0.0049 (13)	0.0035 (11)	−0.0017 (12)
C10	0.0408 (13)	0.0408 (13)	0.0373 (12)	0.0047 (10)	0.0082 (10)	−0.0009 (10)
C11	0.0430 (13)	0.0410 (13)	0.0401 (13)	0.0039 (10)	0.0061 (10)	0.0035 (10)
C12	0.0443 (13)	0.0371 (12)	0.0403 (13)	0.0057 (10)	0.0052 (10)	−0.0052 (10)
C13	0.0579 (16)	0.0540 (16)	0.0456 (15)	0.0029 (13)	0.0033 (12)	0.0047 (12)
C14	0.117 (4)	0.084 (3)	0.086 (3)	0.005 (3)	−0.002 (3)	−0.030 (2)
N1	0.0432 (11)	0.0360 (10)	0.0387 (11)	0.0087 (9)	0.0022 (9)	−0.0041 (8)
N2	0.0381 (10)	0.0326 (10)	0.0431 (11)	0.0030 (8)	0.0069 (8)	−0.0029 (8)
O1	0.0509 (10)	0.0440 (10)	0.0360 (9)	0.0060 (8)	0.0003 (7)	−0.0027 (7)
O2	0.0774 (14)	0.0407 (11)	0.0497 (11)	0.0110 (9)	0.0048 (9)	−0.0083 (8)
O3	0.0373 (9)	0.0340 (9)	0.0655 (11)	−0.0004 (7)	0.0156 (8)	−0.0049 (8)
O4	0.0518 (11)	0.0389 (10)	0.0658 (12)	−0.0061 (8)	0.0198 (9)	−0.0010 (8)
O5	0.0466 (10)	0.0450 (10)	0.0570 (11)	−0.0068 (8)	0.0119 (9)	−0.0017 (8)
S1	0.0590 (5)	0.0937 (7)	0.0592 (5)	0.0013 (4)	−0.0082 (4)	0.0056 (4)

### Geometric parameters (Å, º)

**Table:**

C1—C2	1.510 (4)	C8—H8A	0.9600
C1—H1A	0.9600	C8—H8B	0.9600
C1—H1B	0.9600	C8—H8C	0.9600
C1—H1C	0.9600	C9—O1	1.482 (3)
C2—O3	1.472 (3)	C10—O2	1.210 (3)
C2—C4	1.513 (4)	C10—O1	1.331 (3)
C2—C3	1.514 (4)	C10—N1	1.367 (3)
C3—H3A	0.9600	C11—N1	1.460 (3)
C3—H3B	0.9600	C11—C12	1.505 (4)
C3—H3C	0.9600	C11—H11A	0.9700
C4—H4A	0.9600	C11—H11B	0.9700
C4—H4B	0.9600	C12—O5	1.418 (3)
C4—H4C	0.9600	C12—C13	1.534 (4)
C5—O4	1.204 (3)	C12—H12	0.9800
C5—O3	1.345 (3)	C13—S1	1.794 (3)
C5—N2	1.363 (3)	C13—H13A	0.9700
C6—C9	1.518 (5)	C13—H13B	0.9700
C6—H6A	0.9600	C14—S1	1.769 (5)
C6—H6B	0.9600	C14—H14A	0.9600
C6—H6C	0.9600	C14—H14B	0.9600
C7—C9	1.511 (5)	C14—H14C	0.9600
C7—H7A	0.9600	N1—N2	1.393 (3)
C7—H7B	0.9600	N2—H2	0.9282
C7—H7C	0.9600	O5—H5	0.8200
C8—C9	1.508 (4)		
			
C2—C1—H1A	109.5	H8B—C8—H8C	109.5
C2—C1—H1B	109.5	O1—C9—C8	108.9 (2)
H1A—C1—H1B	109.5	O1—C9—C7	110.5 (2)
C2—C1—H1C	109.5	C8—C9—C7	112.9 (3)
H1A—C1—H1C	109.5	O1—C9—C6	101.2 (2)
H1B—C1—H1C	109.5	C8—C9—C6	111.3 (3)
O3—C2—C1	101.8 (2)	C7—C9—C6	111.4 (3)
O3—C2—C4	109.2 (2)	O2—C10—O1	125.9 (2)
C1—C2—C4	111.8 (3)	O2—C10—N1	122.8 (2)
O3—C2—C3	110.4 (2)	O1—C10—N1	111.3 (2)
C1—C2—C3	110.7 (3)	N1—C11—C12	114.0 (2)
C4—C2—C3	112.3 (3)	N1—C11—H11A	108.7
C2—C3—H3A	109.5	C12—C11—H11A	108.7
C2—C3—H3B	109.5	N1—C11—H11B	108.7
H3A—C3—H3B	109.5	C12—C11—H11B	108.7
C2—C3—H3C	109.5	H11A—C11—H11B	107.6
H3A—C3—H3C	109.5	O5—C12—C11	108.3 (2)
H3B—C3—H3C	109.5	O5—C12—C13	111.4 (2)
C2—C4—H4A	109.5	C11—C12—C13	107.6 (2)
C2—C4—H4B	109.5	O5—C12—H12	109.8
H4A—C4—H4B	109.5	C11—C12—H12	109.8
C2—C4—H4C	109.5	C13—C12—H12	109.8
H4A—C4—H4C	109.5	C12—C13—S1	115.8 (2)
H4B—C4—H4C	109.5	C12—C13—H13A	108.3
O4—C5—O3	127.7 (2)	S1—C13—H13A	108.3
O4—C5—N2	125.6 (2)	C12—C13—H13B	108.3
O3—C5—N2	106.7 (2)	S1—C13—H13B	108.3
C9—C6—H6A	109.5	H13A—C13—H13B	107.4
C9—C6—H6B	109.5	S1—C14—H14A	109.5
H6A—C6—H6B	109.5	S1—C14—H14B	109.5
C9—C6—H6C	109.5	H14A—C14—H14B	109.5
H6A—C6—H6C	109.5	S1—C14—H14C	109.5
H6B—C6—H6C	109.5	H14A—C14—H14C	109.5
C9—C7—H7A	109.5	H14B—C14—H14C	109.5
C9—C7—H7B	109.5	C10—N1—N2	120.5 (2)
H7A—C7—H7B	109.5	C10—N1—C11	119.7 (2)
C9—C7—H7C	109.5	N2—N1—C11	115.85 (19)
H7A—C7—H7C	109.5	C5—N2—N1	119.2 (2)
H7B—C7—H7C	109.5	C5—N2—H2	119.6
C9—C8—H8A	109.5	N1—N2—H2	115.4
C9—C8—H8B	109.5	C10—O1—C9	120.6 (2)
H8A—C8—H8B	109.5	C5—O3—C2	122.31 (19)
C9—C8—H8C	109.5	C12—O5—H5	109.5
H8A—C8—H8C	109.5	C14—S1—C13	101.96 (19)
			
N1—C11—C12—O5	62.4 (3)	C11—N1—N2—C5	92.7 (3)
N1—C11—C12—C13	−177.0 (2)	O2—C10—O1—C9	−9.8 (4)
O5—C12—C13—S1	−71.2 (3)	N1—C10—O1—C9	171.7 (2)
C11—C12—C13—S1	170.10 (19)	C8—C9—O1—C10	72.2 (3)
O2—C10—N1—N2	171.3 (2)	C7—C9—O1—C10	−52.4 (4)
O1—C10—N1—N2	−10.2 (3)	C6—C9—O1—C10	−170.5 (2)
O2—C10—N1—C11	14.5 (4)	O4—C5—O3—C2	7.7 (4)
O1—C10—N1—C11	−167.0 (2)	N2—C5—O3—C2	−174.0 (2)
C12—C11—N1—C10	−141.4 (2)	C1—C2—O3—C5	−179.9 (2)
C12—C11—N1—N2	60.8 (3)	C4—C2—O3—C5	61.8 (3)
O4—C5—N2—N1	−8.6 (4)	C3—C2—O3—C5	−62.3 (3)
O3—C5—N2—N1	173.04 (19)	C12—C13—S1—C14	−89.1 (3)
C10—N1—N2—C5	−65.0 (3)		

### Hydrogen-bond geometry (Å, º)

**Table:**

*D*—H···*A*	*D*—H	H···*A*	*D*···*A*	*D*—H···*A*
O5—H5···O2^i^	0.82	2.03	2.842 (3)	168
N2—H2···O5^i^	0.93	2.07	2.996 (3)	172

Symmetry code: (i) −*x*+1, *y*−1/2, −*z*+1/2.
